# Barodontalgia and its implications for Navy divers and dentists: A narrative review

**DOI:** 10.4317/jced.61619

**Published:** 2024-05-01

**Authors:** Javier García-Torres, Juan J. Segura-Egea

**Affiliations:** 1 DDS. Capt. (OF-2) Spanish Navy, Enfermería B.N. Rota (Clínica Dental), Carretera de Rota s/n, Cadiz, 11530-SPAIN; 2 MD, DDS, PhD. Department of Stomatology (Endodontic Section), School of Dentistry, University of Sevilla, C/ Avicena s/n, 41009-Sevilla, Spain

## Abstract

**Background:**

One of the responsibilities of Spanish Navy Dentists is to carry out dental examinations to military divers. Diving is considered an elite activity and carries a high risk of different pathologies, including barodontalgia which is an oral pain induced by atmospheric pressure changes. This study aims to conduct a narrative review on barodontalgia and its implications for divers and Navy dentists.

**Material and Methods:**

A search was carried out in Pubmed-MEDLINE, Embase and Scielo for all types of articles that explained and related barodontalgia to divers and their diving activities.

**Results:**

Barodontalgia is a rare pathology, but with a higher incidence in the military environment than in the civilian one, being more frequent in upper teeth. Its etiopathogenesis is related to how the tooth reacts to pressure changes. Depending on the time of onset and type of pain during the activity, it can be classified in different ways. The diagnosis is complicated due to the impossibility to reproduce in the dental office the conditions in which it first appeared.

**Conclusions:**

Military dentists must know about barodontalgia and its relationship with other oral pathologies, in order to avoid its appearance in military divers.

** Key words:**Barodontalgia, navy dentists, military divers, scuba diving.

## Introduction

Throughout history, the Spanish Army, Air Force and Navy have been able to coordinate independently all their logistic and medical needs in order to fulfill their assignments set by the Spanish Constitution. In the 80s, with a more specialized and professional Armed Forces, the necessity of creating a common structure that would centralize medical needs in order to meet efficiency and economy criteria, appeared to be desirable.

In 1989, the “Common Corps” of the Spanish Armed Forces was created. This “new structure” within the Ministry of Defense merges four different corps, all being able to serve the Army, Air Force and Navy without distinction. One of these newly created corps was the Military Health Corps which was given different tasks, including preventive, operational and legal tasks.

The Health Corps is composed by physicians, pharmacists, veterinarians, dentists, psychologists and nurses. They are given the rank of First Lieutenant (OF-1) once they leave the Military Academy and can promote to Major General (OF-7), except nurses that can only promote to Lt. Colonel (OF-4).

The members of the Health Corps start their academic training, studying a semester between the three Officers ‘Academies (Army, Air Force and Naval Academies). After this period of time in which they learn the basics to each branch of the Armed Forces, they study another semester in the Military Health Academy in Madrid, where they are taught the specifics of their specialty.

Military dentists are in charge of dental clinics and military personnel´s oral health. Their tasks include dental assessment, application of preventive, legal and forensic dentistry and dental treatment when needed. Other important tasks include deployment tours either on land or on-board naval vessels, under national, NATO or EU mandates.

Probably one of the most important duties for Spanish military dentists are the dental fitness exams. There are different types such as pre-deployment exams, pilot exams, scuba divers’ exams, forensic exams, etc. All results must be in accordance with the “Allied Medical Publication AMedP-4.4, Dental Fitness Standards for Military Personnel and The NATO Dental Fitness Classification System” (1). The main aim of this document is to create a standard regulation for all military dentists from countries belonging to the NATO in order to limit or reduce dental emergencies, especially during international deployments.

All of these examinations are important, but those regarding scuba-diving activities in the Armed Forces have a special consideration and relevance because it is considered to be an elite activity (2). These scuba divers are tasked missions of combat, demolition, artefact destruction, search and rescue, etc.

It is of the utmost importance to assure the physical fitness of those who will participate in scuba-diving activities (3). While doing this activity, different risks can be undergone, such as currents, temperature, animal presence (sharks i.e.), disorientation, low visibility, and one of the most important, decompressive syndromes (4).

This syndrome refers to the accumulation of inert gases in the tissues (5). When ascending, these gases tend to disappear but, at the same time, the pressure level reduces causing these gases to form bubbles that expand. These expanded bubbles can cause a gaseous arterial embolism that can lead to cardiopulmonary arrest, brain damage and tetraplegia (6).

The treatment of this syndrome includes decubitus supine position to favor the denitrogenating of the tissues (5). It is also recommendable to undergo a Hyperbaric Oxygen therapy in a hyperbaric Chamber (7). With this treatment, the patient suffers a new compression with high pressure, leading to the bubbles in the tissues to disappear and be easily be eliminated through breathing. With the oxygen therapy, the affected tissue can easily recover from the damage suffered previously (8).

To try to avoid all possible medical complications, special physical tests are mandatory four scuba divers in the Spanish Armed Forces (9). These tests include an exhaustive oral examination. In order to be declared dentally fit for scuba diving, none of the following conditions are to exist (10).

• Active decay, defective or temporary fillings.

• Inflammatory processes or oral infections.

• Any condition such as malocclusion, removable protheses, etc. that does not allow a correct use of mouthpieces.

• Oral surgery in the previous four weeks.

Even though these examinations are very exquisite, we can never discard the possibility of dental problems during scuba diving. One of the most frequent problems is the barodontalgia which is an oral pain induced by atmospheric pressure changes. It is a consequence of the pulp not being able to adequate its internal pressure when these phenomena occur (11).

To fully understand barodontalgia, it is necessary to know the whole process including its epidemiology, etiopathogenesis, physiopathology, classification and diagnosis. With this information, Navy dentists can get a complete picture of the process that leads up to this phenomenon in order to be able to not only treat it, but also avoid it. This study aims to conduct a narrative review on barodontalgia and its implications for divers and Navy dentists.

## Material and Methods

A search in Pubmed-MEDLINE, Embase and Scielo was conducted in order to find as many articles as possible that versed about barodontalgia and its relationship to scuba diving. The key words to the search were barodontalgia, scuba diving and divers (“barodontalgia”(All Fields) AND ((“scuba”(All Fields) AND (“dived”(All Fields) OR “dives”(All Fields) OR “diving”(MeSH Terms) OR “diving”(All Fields) OR “divings”(All Fields))) OR (“diver”(All Fields) OR “diver s”(All Fields) OR “divers”(All Fields)))). The inclusion criteria for articles were the fact that they wrote about the epidemiology, etiopathogenesis, physiopathology, classification and diagnosis of barodontalgia. Also, all articles about barodontalgia among divers, military or not, or case reports were accepted. A total of 61 articles were obtained. After reading the titles and abstracts, a total of 22 articles that met the criteria described in the previous paragraph were selected.

Results and Discussion

-Barodontalgia: Concept and epidemiology

There is still a lot of controversy around the concept of barodontalgia. It is a pathology with a low incidence in the civilian world whereas in the military world, the incidence rises (12). This is a consequence of the low interest it awakens for civilian dentists in comparison to military dentists whom find it very interesting due to the implications barodontalgia can have in the population they attend to.

Barodontalgia is a pathology that has a low prevalence when studied in hyperbaric chambers, between 1% (13) and 2% (14). In scuba diving this prevalence rises up to 5-7% (15), and amongst military divers it can be as high as 17% (16).

The incidence of barodontalgia is higher (between 48-56%) in upper teeth than in lower teeth (29-32%) (13,17). First upper molars and first lower molars are the most affected (13,18).

-Etiopathogenesis

**Figure 1:**
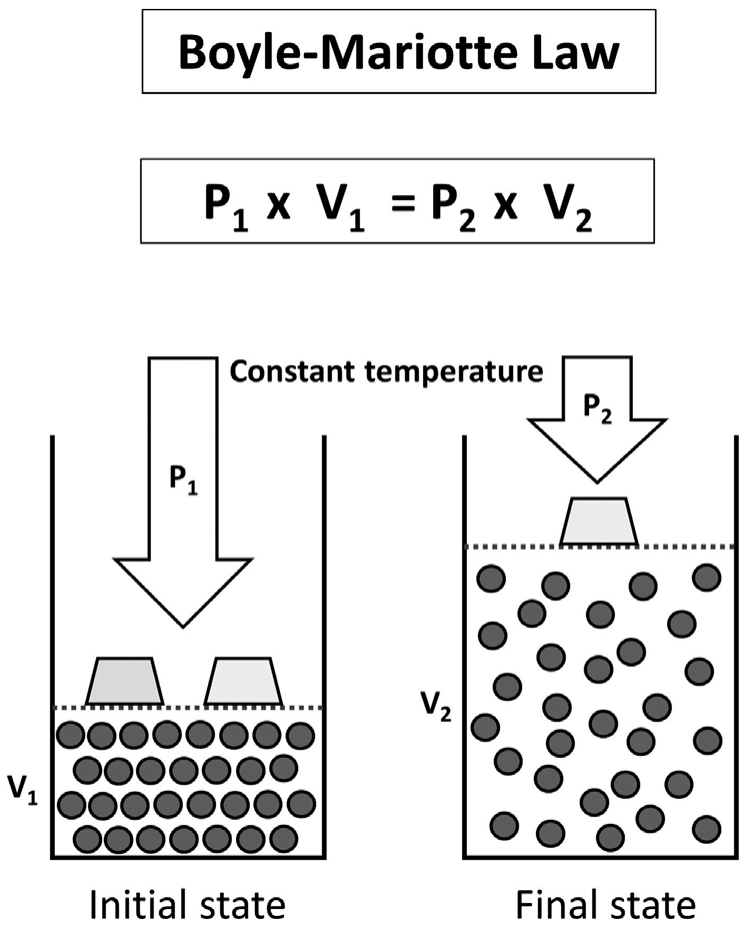
Boyle-Mariotte law. The absolute pressure exerted by a given mass of an ideal gas is inversely proportional to the volume it occupies if the temperature and amount of gas remain unchanged within a closed system.

The etiopathogenesis is still a controversy because the mechanism through which it is caused is not yet clear (15,19). Kollman proposed three theories based on histological findings (11,18,20). The most accepted one amongst the three theories was the one based on Boyle´s Law that states that at certain temperature, the volume of a gas is inversely proportional to the atmospherics’ pressure (Fig. 1).

If a filling or root canal has an air bubble trapped inside, when exposed to pressure changes, this bubble will expand or contract activating nociceptors producing pain (21).

In scuba diving there are two mechanisms that can produce barodontalgia (20). Firstly, during the descent the air breathed can penetrate the tooth (through decays or deficient restorations i.e.), it is compressed and pushed to the pulp or dentinal tubules. The second mechanism occurs ascending, when air trapped expands because the external pressure decreases. This air is not able to evacuate as fast as it came in leading to pain or even tooth or restoration fracture.

-Symptoms and clinical classification

Different classifications for barodontalgia have been suggested. It can be classified regarding its origin, being direct when the origin is periapical or dental or indirect when the origin is different, such a barosinusitis (22,23). However, the most used classification is based on the origin and symptoms developed as summarized in the next graphic (24) ( Table 1).

If pain appears when ascending it can indicate pulp vitality with evidence of irreversible pulpitis. If it appears descending it can indicate pulpar necrosis (24,25). When there is perirradicular pathology, the pain can appear either ascending or descending (13,24). In most cases, the pain disappears when the pressure reaches its initial state, except in perirradicular pathology cases in which the pain can last longer (13,14,24,26).

-Diagnosis of barodontalgia 

Diagnosing barodontalgia is a complicated quest as it is a consequence of a preexistent pathology and it is almost impossible for dentist to reproduce the conditions in which it appeared in the first place (19,23). This type of pain can be confused with other types of dental pain such as that derived from carious or pulp lesions, further complicating the diagnosis.

Dentists have to rely on performing a deep clinical record to observe certain issues related to the appearance of barodontalgia, such as defective fillings which can cause up to 84% of barodontalgia cases (24). In this detailed clinical evaluation, dental sensitivity tests, x-rays, percussion tests and even additional complementary tests such as computed tomography must be performed to make an accurate differential diagnosis with pathologies that cause similar pain. Thus, it is important to rule out other possible causes of dental pain such as cavities, periodontal disease, problems in the temporomandibular joint, etc (11).

It is also important to conduct a good questionnaire to obtain every detail and all the information about the pain suffered so that, if the pain has been diagnosed as barodontalgia, the dentist is able to apply the classification shown in Table 1. Once the pain has been classified and related to a possible cause, the treatment will be more accurate.

As it can be seen, few studies have been conducted regarding barodontalgia, most of them in different armed forces (12,13,15,16,27). This is the reason why there is no specific criteria or guidelines for dentist to apply in the few cases they might encounter in their practice.

Prevention of barodontalgia

The International Dental Federation (FDI) recommends annual checkups for scuba divers (28). The reason for these checkups is to define any possible condition that would cause a problem when a changing pressure situation is undergone by the patient. In addition to this, a few other recommendations are useful for the dentist to follow when it comes to scuba diving patients:

- After a treatment with anesthesia, scuba diving is not recommended in the first 24 hours (27).

- After an oral surgery, diving activities must cease for at least a week. After tooth extractions, it is of the utmost importance to disregard oral-sinus communications. In this case, no scuba diving should be authorized for at least two weeks (27,28).

- Scuba diving with provisional restorations or provisionally cemented crowns is not recommended due to the risk of decementation and aspiration. Definitive restorations and crown cementation are to be completed before the activity (20,29).

- All decays must be eliminated and properly restored. If there is pulp exposition, a root canal must be done (29). If the cavity is deep, cavity protectors such as glass ionomer cement can be applied (23).

- Removable prothesis can be aspired during the activity leading to death (29), therefore it must be removed before scuba diving.

- Periodontal disease must be treated and under control (29).

- Root canal treatments are to be completed before scuba diving. Initiated and unfinished root canal treatments can lead to odontocresis (20).

- If due to special circumstances, especially in the military, a definitive treatment cannot be finished prior to the start of the activity, tooth extraction is the treatment of choice.

-Treatment of barodontalgia

As it has been explained, the exact causes that produce this pathology are still not known exactly, so the treatment that must be carried out is defined by the pathologies that can lead to barodontalgia. Pulp exposures and recent restorative treatments have been described as the most common causes of barodontalgia (24).

The scientific evidence associates barodontalgia with cavities or defective restorations at the dentin level, so the treatment of these patients must include the correct elimination of the caries and its adequate filling (11). For this, the material of choice will be resin cement (29). In cases of deep restorations, it is advisable to apply a cavity base or pulp protector such as glass ionomer cement (23,29).

When pulp exposure is suspected or exists in personnel subjected to pressure changes, direct pulp capping is contraindicated (19,29). In these cases, the indicated treatment will be root canal treatment with hot gutta-percha, preferably in a single session since this eliminates the risk of air trapping in the spaces, both in the root canals and in the provisional restoration (23).

In military divers where their activity is necessary to fulfill a mission and they are in one of the situations described that can cause barodontalgia, the administration of a combination of non-steroidal anti-inflammatory drugs (NSAIDs) with another non-NSAID analgesic such as paracetamol is recommended (30).

## Conclusions

There is still a long path ahead regarding barodontalgia. Even though it is a pathology with a low prevalence, its importance is fundamental, especially for dentists in the armed forces. Studies have merely scratched the surface of a concept that has implications in all branches of dental medicine, including restorative dentistry, endodontics, prosthetics and periodontology.

## Figures and Tables

**Table 1:** Barodontalgia classification according to Ferjentsik et al. (1982).

Class	Cause	Symptoms
Class I	Irreversible pulpitis	Acute pain on ascent
Class II	Reversible pulpitis	Dull pain on ascent
Class III	Necrotic pulp	Dull pain on descent
Class IV	Periapical pathology	Persistent severe pain on ascent or descent

## Data Availability

The datasets used and/or analyzed during the current study are available from the corresponding author.
